# Multi‐omics characterization of lymphedema‐induced adipose tissue resulting from breast cancer‐related surgery

**DOI:** 10.1096/fj.202400498RR

**Published:** 2024-10-12

**Authors:** Sinem Karaman, Satu Lehti, Cheng Zhang, Marja‐Riitta Taskinen, Reijo Käkelä, Adil Mardinoglu, Håkan Brorson, Kari Alitalo, Riikka Kivelä

**Affiliations:** ^1^ Wihuri Research Institute Helsinki Finland; ^2^ Translational Cancer Medicine Research Program Faculty of Medicine, University of Helsinki Helsinki Finland; ^3^ Individualized Drug Therapy Research Program Faculty of Medicine, University of Helsinki Helsinki Finland; ^4^ Faculty of Sport and Health Sciences University of Jyväskylä Jyväskylä Finland; ^5^ Science for Life Laboratory KTH‐Royal Institute of Technology Stockholm Sweden; ^6^ Research Programs Unit, Clinical and Molecular Metabolism University of Helsinki Helsinki Finland; ^7^ Helsinki University Lipidomics Unit (HiLIPID) Helsinki Institute of Life Science (HiLIFE) and Biocenter Finland Helsinki Finland; ^8^ Molecular and Integrative Biosciences Research Program Faculty of Biological and Environmental Sciences, University of Helsinki Helsinki Finland; ^9^ Centre for Host‐Microbiome Interactions Faculty of Dentistry, Oral & Craniofacial Sciences, King's College London London UK; ^10^ Department of Clinical Sciences in Malmö Lund University Malmö Sweden; ^11^ Plastic and Reconstructive Surgery Skåne University Hospital Malmö Sweden; ^12^ Lund University Cancer Centre Lund Sweden; ^13^ Department of Health Sciences, Faculty of Medicine, Health & Human Sciences Macquarie University Sydney New South Wales Australia; ^14^ Stem Cell and Metabolism Research Program Faculty of Medicine, University of Helsinki Helsinki Finland

**Keywords:** adipose‐deposition, lipidomics, liposuction, lymphedema, metabolomics, transcriptomics

## Abstract

Secondary lymphedema (LE) following breast cancer‐related surgery is a life‐long complication, which currently has no cure. LE induces significant regional adipose tissue deposition, requiring liposuction as a treatment. Here, we aimed to elucidate the transcriptional, metabolomic, and lipidomic signature of the adipose tissue developed due to the surgery‐induced LE in short‐ and long‐term LE patients and compared the transcriptomic landscape of LE adipose tissue to the obesity‐induced adipose tissue. Adipose tissue biopsies were obtained from breast cancer‐operated females with LE from the affected and non‐affected arms (*n* = 20 patients). To decipher the molecular properties of the LE adipose tissue, we performed RNA sequencing, metabolomics, and lipidomics combined with bioinformatics analyses. Differential gene expression data from a cohort of lean and obese patients without LE was used for comparisons. Integrative analysis of functional genomics revealed that inflammatory response, cell chemotaxis, and angiogenesis were upregulated biological processes in the LE arm, indicating a sustained inflammation in the edematous adipose tissue; whereas, epidermal differentiation, cell–cell junction organization, water homeostasis, and neurogenesis were downregulated in the LE arm. Surprisingly, only a few genes were found to be the same in the LE‐induced and the obesity‐induced adipose tissue expansion, indicating a different type of adipose tissue development in these two conditions. In metabolomics analysis, we found reduced levels of a branched‐chain amino acid valine in the LE arm and downregulation of the mRNA levels of its transporter *SLC6A15*. Lipidomics analyses did not show any significant differences between the LE and non‐LE arms, suggesting that other factors affect the lipid composition of the adipose tissue more than the LE in these patients. Our results provide a detailed molecular characterization of adipose tissue in secondary LE after breast cancer‐related surgery. We also show distinct differences in transcriptomic signatures between LE‐induced adipose tissue and obesity‐induced adipose tissue, but only minor differences in metabolome and lipidome between the LE and the non‐LE arm.

## Graphical abstract

This study analyzes adipose tissue changes using RNA sequencing, metabolomics, and lipidomics in lymphedema after breast cancer‐related surgery. Transcriptomics analyses revealed increased inflammation, new blood vessel formation, and reduced skin cell and nerve growth in lymphedema adipose tissue. Compared to obesity, only a few genes were similarly affected. Additionally, lower levels of the amino acid valine and its transporter were observed in lymphedema adipose tissue, while lipid composition remained similar in both swollen and nonswollen arms.

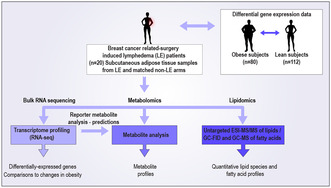

**Abbreviations**
CDT: complete decongestive therapy
DEGs: differentially‐expressed genes
FA: fatty‐acid
GEMs: genome scale metabolic models
GO: gene ontology
LE: lymphedema
PC: phosphatidylcholine
PC1: principal component 1
PC2: principal component 2
PCA: principal component analysis
SM: sphingomyelin
TAG: triacylyglycerol

## 1 INTRODUCTION

Lymphedema (LE) is an excessive accumulation of a protein‐rich extracellular fluid (lymph) in the interstitial compartment due to defective lymphatic function (reviewed in^1^, ^2^). The accumulation of fluid elicits an inflammatory reaction, induces fibrosis and adipose tissue accumulation, and leads to impaired wound healing and immune responses.^3^, ^4^ The prevalence estimates of chronic LE are between 1.33 and 1.44 per 1000 individuals.^5^, ^6^, ^7^ However, unreported cases are common, thus it is thought that the reported prevalence is an underestimate.^7^ LE is categorized into two subclasses according to the underlying pathophysiology: primary LE, also known as hereditary LE, and secondary (acquired) LE.^1^ While primary LE is congenital, secondary LE is caused by filariasis or obliteration of the lymphatic vasculature or lymph nodes by trauma, surgery, radiation therapy, or infection.^3^, ^8^

Secondary LE is more common, and its pathophysiology has been more comprehensively studied (reviewed in^9^, ^10^, ^11^). Breast cancer‐related LE is the most common form of LE in developed countries,^12^ predominantly related to cancer treatment: the surgical removal of lymph nodes and radiotherapy. In the aftermath of the surgical procedure to remove the lymph nodes, which is often followed by irradiation to kill remaining tumor cells, the lymphatic vasculature is commonly damaged beyond repair. This initially leads to a dilation of the lymphatic vasculature, followed by increased lymphatic endothelial cell proliferation but ultimately leads to lymphatic valve malfunction, fibrosis of smooth muscle cells in the walls of the lymphatics, and LE. To date, there is no universally effective treatment or cure for LE. The available treatment approaches aim at reducing the symptoms and mostly rely on physiotherapy, complete decongestive therapy (CDT) comprising of manual drainage, bandaging, exercises, flat‐knitted compression garments, and skin care^13^ or controlled compression therapy by restrictive bandaging.^14^ Surgical alternatives include liposuction of the edematous limb^14^, ^15^, ^16^, ^17^, ^18^ and microvascular reconstruction, involving lymphatico‐venous or lymphatico‐venous‐lymphatic shunts,^19^, ^20^, ^21^, ^22^, ^23^, ^24^ lymph node transfer,^25^, ^26^, ^27^ or lymph vessel transplantation.^28^, ^29^, ^30^, ^31^

LE patients often present with local adipose tissue overgrowth (reviewed in^1^, ^2^). Arm LE is a common complication after surgical removal of axillary lymph nodes for breast cancer treatment.^32^, ^33^, ^34^, ^35^ Neither CDT nor microsurgical reconstruction can be used in later stages of LE as none of the techniques can remove the hypertrophied adipose tissue that occurs in response to lymph stasis and inflammation.^32^, ^33^, ^35^ In later stages of nonpitting LE, which does not respond to conservative treatment, liposuction, combined with postoperative CDT, gives a complete reduction of the excess volume.^14^, ^15^, ^16^, ^17^, ^18^

Increased adiposity in mouse models of obesity has been shown to result in lymphatic dysfunction.^36^, ^37^ On the other hand, LE and leaky lymphatic vessels have been shown to induce adipogenesis and adipose tissue accumulation also in mouse models.^38^, ^39^ Adipogenesis in response to lymphatic fluid stasis is associated with a marked mononuclear cell inflammatory response and upregulation of the expression of adipocyte differentiation markers.^40^ It seems that the underlying pathophysiology of LE can drive adipose‐derived stem cells toward adipogenic differentiation,^41^ but studies have also shown that LE‐induced adipocyte growth occurs via adipocyte hypertrophy.^42^, ^43^ These data support the clinical finding that leaky lymphatics and lymph stasis in LE patients can result in adipose tissue accumulation in the affected region.

The factors involved in LE‐induced adipose tissue accumulation are poorly known. Furthermore, it is not known if adipose tissue growth in LE and obesity follow similar mechanisms. So far, the treatments are only aimed at alleviating LE symptoms, without targeting the underlying causes. Thus, we hypothesized that the adipose tissue of the LE arm has a different molecular composition than the non‐LE arm. We performed transcriptomic, metabolomic, and lipidomic analyses to test this hypothesis and obtain an unbiased insight into the composition of the adipose tissue of the LE‐induced adipose tissue compared to the non‐LE adipose tissue in human patients. We found that the transcriptomic landscape of the LE‐induced adipose tissue was highly different from that of the non‐LE arm, and it markedly differed from obesity‐induced adipose tissue. In contrast, the metabolomic and lipidomic profile of the adipose tissue in the LE arm did not differ much from the healthy arm, suggesting that the individual characteristics and systemic pool of metabolites may be stronger determinants of the metabolome and lipidome of the local adipose tissue than the disease.

## 2 MATERIALS AND METHODS

### 2.1 Patients and sample collection, consent, and ethical permit

Subcutaneous adipose tissue was obtained from the arms of 20 patients undergoing liposuction for chronic lymphedema induced by the surgical treatment for breast cancer. Control subcutaneous adipose tissue was collected from the unaffected arm (non‐LE) of the same individuals. All patients were given general anesthetics, and before the start of liposuction, an adipose tissue biopsy was taken after a 1.5‐cm incision on the medial aspect of the elbow of the normal and lymphedematous arm without the administration of local analgesia. Duration of LE was determined as the time from surgery to liposuction, and the patients were divided into short‐ (≤3 years) and long‐term (≥14 years) LE groups (*n* = 10 patients for transcriptomics and metabolomics and 7 for lipidomics analyses in the short‐term group and 10 for transcriptomics and metabolomics and 9 for lipidomics analyses in the long‐term group). The reason for dividing the patients into two groups with different durations was to see if there were differences in molecular level outcomes as it has been shown that deposition of adipose tissue in lymphedema starts already at the same time when the lymphedema appears (~21 months in average for the first appearance of LE), and by the time it possibly ceases.^33^ The work described has been carried out in accordance with The Code of Ethics of the World Medical Association (Declaration of Helsinki) and informed consent was obtained for tissue collection. The ethical permit for the collection of patient samples is held by Dr. Håkan Brorson and was approved by the Regional Ethical Review Board in Lund, Lund, Sweden (503/2006, 45/2011).

### 2.2 RNA sequencing and analysis

Total RNA was extracted from 20 LE and 20 non‐LE adipose tissue samples using Trizol and purified with an RNeasy Mini kit (Qiagen). Total RNA was subjected to quality control with Agilent Bioanalyzer according to the manufacturer's instructions. To construct libraries suitable for Illumina sequencing the Illumina TruSeq Stranded mRNA Sample preparation protocol which includes cDNA synthesis, ligation of adapters, and amplification of indexed libraries was used. The yield and quality of the amplified libraries were analyzed using Qubit by Thermo Fisher and the Agilent Tapestation. The indexed cDNA libraries were normalized and combined, and the pools were sequenced on the Illumina HiSeq 2000 for a 50‐cycle v3 sequencing run generating 50 bp single‐end reads. Basecalling and demultiplexing were performed using CASAVA software with default settings generating Fastq files for further downstream mapping and analysis. Raw counts were aligned using STAR and counting the reads in gene bodies was defined using a UCSC gtf file. The counts were normalized to counts per million and after TMM normalization in the EdgeR package. Differential expression was analyzed using the DEseq2 package in R.

Venn diagrams comparing the significantly changed transcripts in short‐ and long‐term LE patients were made using the online Venn diagram tool at http://bioinformatics.psb.ugent.be/webtools/Venn/. ShinyGO was used to analyze gene ontologies and functional genomics from the RNAseq data (http://bioinformatics.sdstate.edu/go/).

### 2.3 Quantitative real‐time PCR

RNA was extracted from frozen adipose tissue pieces (50–100 mg) with the RNeasy Lipid tissue Mini Kit (Qiagen) following the manufacturer's instructions. cDNA was transcribed from a 500 ng RNA template, using the high‐capacity reverse transcription kit (Applied Biosystems). The RT‐qPCR was performed using a BIO‐RAD C1000 Thermal cycler according to a standardized protocol (BIO‐RAD), with Roche SYBR green. *RPLP0* (*36B4*) was used as internal control and fold changes of gene expression were calculated using the DeltaDeltaCt method and compared with a paired t‐test. The primer sequences used can be found in Table S1.

### 2.4 Reporter metabolite analysis

Reporter metabolite analysis was run on the data from RNA seq using a previously published pipeline.^44^ In brief, this method retrieves a gene‐metabolite network from the genome‐scale metabolic models (GEMs) and identifies key metabolite hubs with the outstanding amount of DE genes that are obtained as described above directly connecting to them. Previously published high‐quality GEMs for adipose tissue^45^ were used as input for the reporter metabolite analysis.

### 2.5 Comparison to obesity dataset

The differentially expressed genes (DEGs) in white adipose tissue obtained from non‐LE lean subjects and subjects with obesity (all females) without LE were retrieved from a previous study.^45^ This gene list was compared to the DEGs in our dataset comparing the LE and non‐LE adipose tissues to reveal if obesity‐induced adipose tissue enlargement results in similar changes in adipose tissue transcriptome as LE. The enriched gene ontology (GO) term network of the overlapped genes was visualized using cytoscape with the external package EnrichmentMap.^46^

### 2.6 Metabolomics

Metabolomics analyses were carried out at the Biocenter Finland and HiLIFE‐supported Metabolomics Unit, Institute for Molecular Medicine Finland FIMM, HiLIFE, University of Helsinki. Approximately 20 mg of frozen adipose tissue per sample (*n* = 20 + 20) was analyzed and normalized to the exact weight according to the method described.^47^

### 2.7 Lipidomics

Seven pairs of samples from short‐term LE and nine pairs of samples from long‐term LE had a sufficient amount of sample available for lipidomic analysis (*n* = 16 LE +16 non‐LE samples). Lipidomic analyses of adipose tissue pieces (50–100 mg) included shotgun electrospray ionization tandem mass spectrometry (ESI‐MS/MS) of triacylglycerol (TAG), phosphatidylcholine (PC) and sphingomyelin (SM) species, and gas chromatography of fatty acids (FA) (FID and MS detections for quantification and structure confirmation, respectively). These analyses were carried out as described,^48^ except, in the present work, the equipment recording electron impact mass spectra for FA structure confirmation was Shimadzu GCMS‐QP2010 Ultra (Shimadzu Scientific Instruments, Kyoto, Japan).

### 2.8 Analysis of lipidomics data

Instead of separately presenting fold change values for the relative concentrations of numerous individual FA and lipid species in adipose tissues from the LE and non‐LE arms, the FA compositions in total lipids and lipid species compositions in each lipid class were compared by principal component analysis (PCA). The magnitude of the compositional difference on PC1 and PC2 scores was normalized to the average compositional difference of the two arms in the 16 individuals and illustrated in heatmap format.

## 3 RESULTS

Paired subcutaneous adipose tissue samples were collected from both forearms of 20 breast cancer patients who had developed unilateral post‐surgical LE. The patients were divided into short and long‐term LE groups (short ≤3 years and long ≥14 years, respectively). The collected adipose tissue samples were then used for transcriptomic, metabolomic, and lipidomic analyses. The patient demographic data and clinical characteristics of the patients with breast cancer surgery‐related LE are shown in Table 1.

**TABLE 1:** Demographic data of the breast cancer surgery‐related lymphedema patients.

Lymphedema category	Affected arm	Age at biopsy (years± SD)	Gender (female/total)	Cancer surgery to LE start (years ± SD)	LE duration (years ± SD)	BMI	Erysipleas occurrence (yes/total)
All (*n* = 20)	Right (*n* = 9) Left (*n* = 11)	64.1 ± 11.7	20/20	2.2 ± 2.7	12.7 ± 9.0	28.6 ± 3.2	10/20
Short (≤3 years) (*n* = 10)	Right (*n* = 4) Left (*n* = 6)	60.8 ± 14.3	10/10	2.7 ± 3.4	2.2 ± 0.6	29.6 ± 3.7	2/10
Long (≥14 years) (*n* = 10)	Right (n = 5) Left (*n* = 5)	67.4 ± 7.7	10/10	1.6 ± 1.8	18.8 ± 5.6	27.6 ± 2.4	8/10

### 3.1 Altered adipose tissue gene signature in LE

**FIGURE 1:**
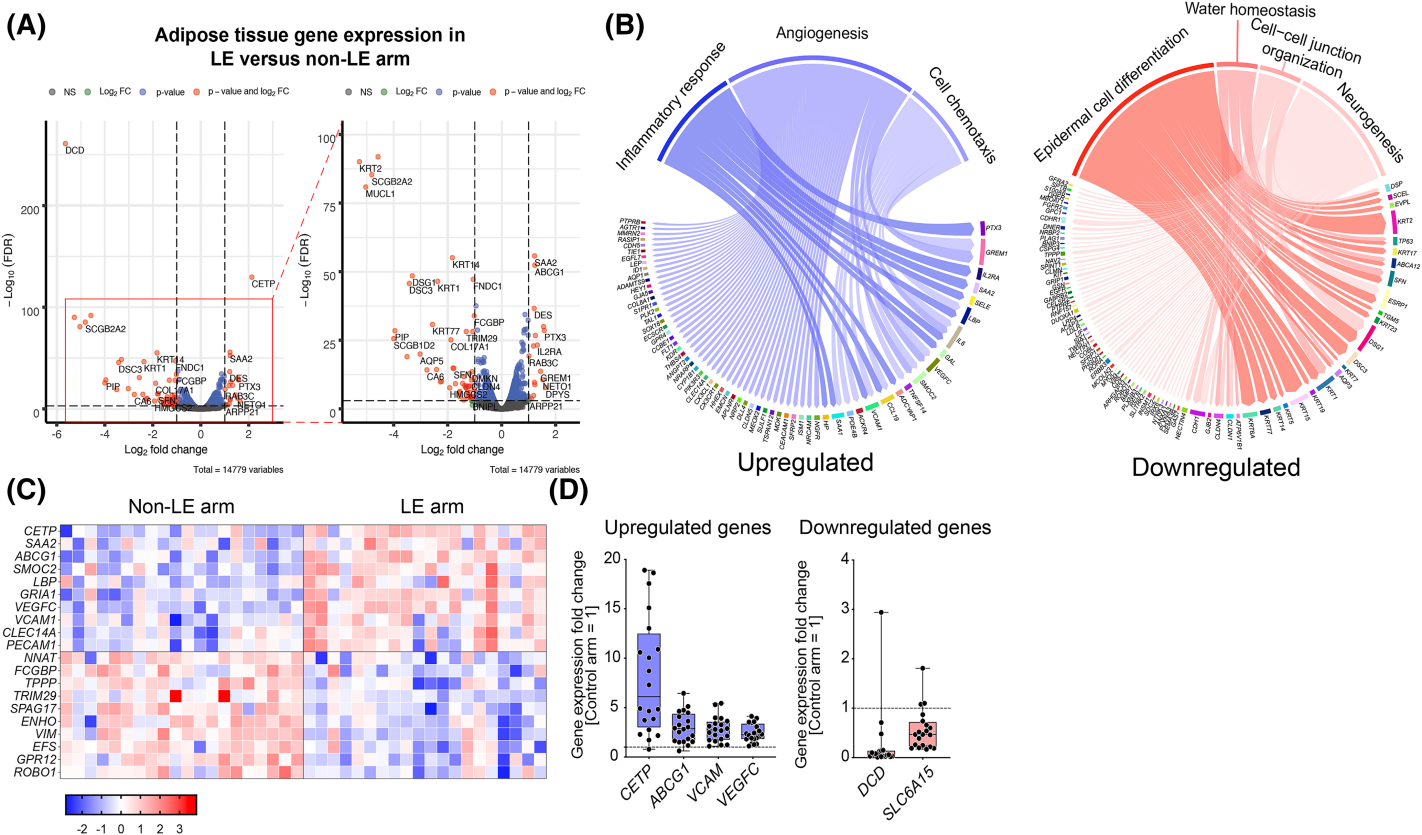
RNA sequencing of adipose tissue from the lymphedema and the control arms. (A) Volcano plots of all differentially expressed genes between the LE arm and the control (non‐LE) arm (FDR cut‐off = 0.001, log_10_ FC <−0.25 or >0.25, *n* = 20), and a close‐up for the transcripts shown in the red rectangle. (B) Significantly up and downregulated GO terms and selected genes within the GO terms suggest an upregulation in inflammatory response, angiogenesis, and cell chemotaxis pathways, whereas epidermal cell differentiation, water homeostasis, cell–cell junction organization, and neurogenesis pathways were significantly downregulated. (C) Heatmap of selected genes up‐ or downregulated in the lymphedema arm compared to the control arm. (D) qPCR validation of selected genes. Dashed line indicates the expression level in the control arm (=1).

To obtain an unbiased insight into the transcriptional changes associated with LE in adipose tissue of the lymphedematous arm, we performed global gene expression analyses with RNA sequencing using the non‐LE arm as a control. We identified a total of 1473 transcripts that were dysregulated in all LE patients, of which 830 were upregulated and 643 downregulated (Table S2). The most upregulated genes in the LE arm included genes related to cholesterol metabolism (e.g., *CETP*, *ABCG1*) and to inflammation (e.g., *VEGFC*, *VCAM1*; Figure 1A). Cholesteryl ester transfer protein (*CETP*), a plasma protein involved in cholesteryl ester and TAG transfer between LDL and HDL, was the most significantly upregulated gene in LE. The most significantly repressed gene was dermcidin (*DCD*), an important antimicrobial gene produced by the sweat glands of the skin.^49^ Among the most downregulated genes was also *SLC6A15*, a branched‐chain amino acid transporter. In addition, several keratins were downregulated in the adipose tissue samples from the LE arms.

GO enrichment analysis of the upregulated genes showed that inflammatory response, cell chemotaxis, and angiogenesis were among the most affected biological processes in the adipose tissue from the LE arm, indicating a sustained inflammation that may increase blood flow and lymph formation in the edematous adipose tissue (Figure 1B). Genes related to epidermal differentiation, cell–cell junction organization, water homeostasis, and neurogenesis were found to be repressed in the LE versus non‐LE arm (Figure 1B). Heatmap of selected genes' Z‐scores and the validation of gene expression changes with qPCR are shown in Figure 1C,D, respectively.

### 3.2 Different transcriptomic landscapes in short‐term and long‐term LE

**FIGURE 2:**
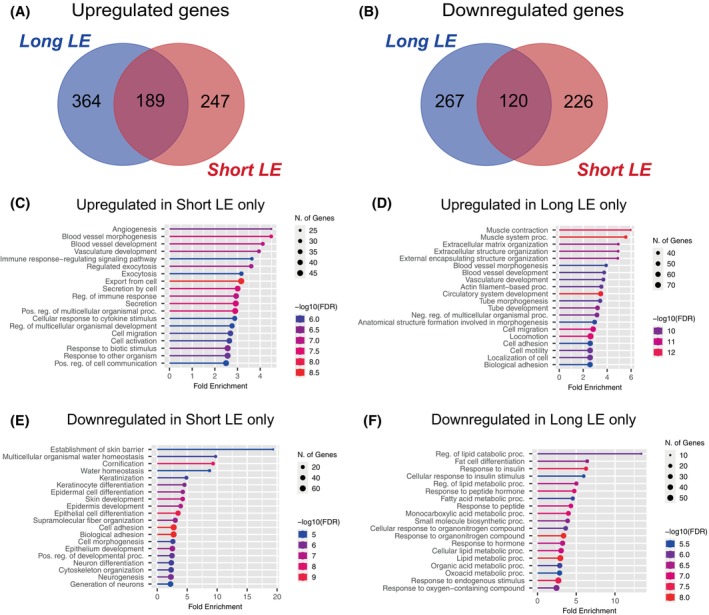
Comparison of the short‐term and long‐term LE. (A and B) Venn diagrams showing genes significantly up (A) or downregulated (B) in short‐term (≤3 years) and long‐term (≥14 years) LE (FDR cut‐off = 0.01, log_10_ FC <−0.25 or >0.25, *n* = 10 versus 10). (C) GO term analysis of the 247 genes upregulated only during short‐term LE. (D) GO term analysis of the 364 genes upregulated only during long‐term LE. (E) GO term analysis of the 226 genes downregulated only during short‐term LE. (F) GO term analysis of the 267 genes downregulated only during long‐term LE.

Next, we studied if the LE adipose tissue transcriptome is affected by the duration of the disease. The patients were divided into short and long‐term LE groups. Comparison of the differentially expressed genes between these subgroups demonstrated an overlap in both up and downregulated genes between the groups, but also a considerable number of genes unique for each group. This indicates that the transcriptomic landscape in LE‐derived adipose tissue continues to evolve after the initial development of the disease. The Venn diagrams comparing the groups are shown in Figure 2A,B. Gene ontology analysis of the genes unique for each subgroup revealed that in the short‐term LE group, angiogenesis, vasculature development, and immune response were the most upregulated pathways (Figure 2C), indicating an immune response and an attempt to restore blood and lymphatic circulation. In the long‐term LE group, muscle contraction and extracellular matrix organization were the most activated pathways, followed by vasculature development (Figure 2D). The most repressed pathways in the short‐term LE were the establishment of skin barrier, water homeostasis, keratinization, and neurogenesis (Figure 2E), whereas, in the long‐term LE tissues, lipid metabolism, fat cell differentiation, and response to insulin were highly downregulated (Figure 2F).

### 3.3 Comparison of adipose tissues in LE and obesity

To study if LE‐induced adipose tissue expansion results in similar changes in adipose tissue transcriptomic landscape, we compared the differentially expressed genes (DEGs) in our dataset to DEGs found in obesity‐induced adipose tissue growth. For this, we used data from a previously published cohort comparing subcutaneous adipose tissue of female subjects with obesity (BMI ≥30) to lean subjects (*n* = 80 with obesity and *n* = 112 lean controls).^45^

**FIGURE 3:**
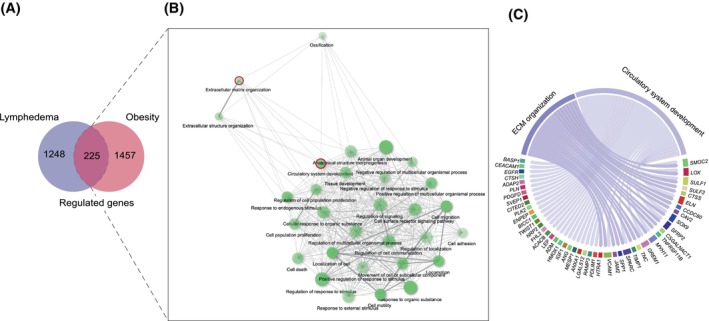
Differential transcriptomic changes during the subcutaneous adipose tissue enlargement in lymphedema versus obesity. (A) A Venn diagram showing the number of affected genes in LE‐versus non‐LE adipose tissue compared to adipose tissue from obese versus lean subjects. Only 225 genes were significantly co‐regulated in these two conditions. (B) GO term networks of the 225 genes. (C) Genes that fall under the GO term extracellular matrix (ECM) organization and circulatory system development.

Surprisingly, we found relatively little overlap between adipose tissue transcriptomic changes induced by obesity versus LE, as out of 1682 differentially expressed genes (DEG) between obese and lean subjects and 1473 DEGs between the LE and non‐LE arm, only 225 genes were shared between the two conditions (Figure 3A, Table S3). This suggested major differences in the development and characteristics of these two modes of adipose tissue expansion. The GO enrichment analysis showed that the genes common to both conditions were related to extracellular matrix organization, cell proliferation and migration, and circulatory system development (Figure 3B,C).

### 3.4 Reporter metabolite predictions and metabolomics results

**FIGURE 4:**
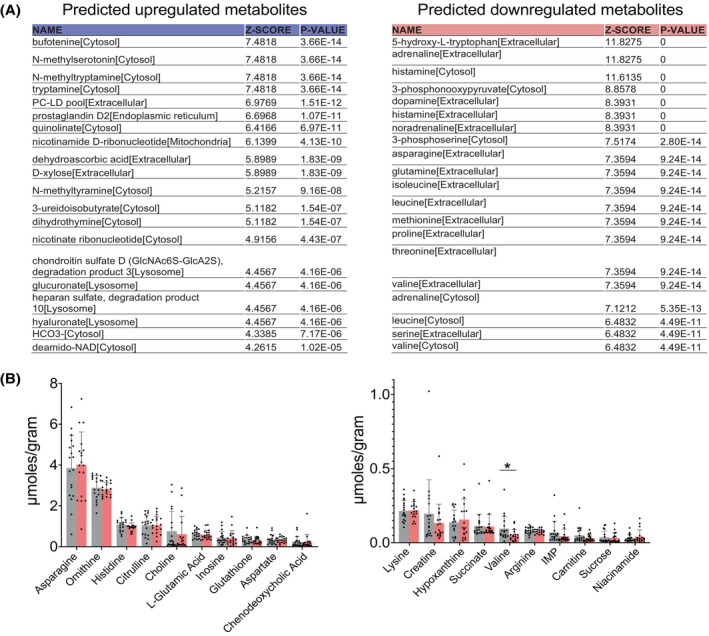
Predicted reporter metabolites and metabolomics analysis. (A) Top 20 predicted upregulated and downregulated reporter metabolites based on the RNA sequencing data suggest upregulation of nucleoside metabolism and downregulation of amino acid metabolism in the LE adipose tissue. (B) Targeted analysis of metabolites between the adipose tissues of control (non‐LE) and LE arm of the same patient shows mostly unchanged metabolites with the exception of lower valine levels (*FDR <0.05).

In order to have a better understanding of potential changes in metabolites, we performed a reporter metabolite analysis on the LE versus non‐LE arm differentially expressed genes by using the RNA sequencing data as input, as previously described.^44^ Based on the changes in gene expression, this analysis predicted an upregulation of nucleotide metabolism and a downregulation of amino acid metabolism including branched‐chain amino acids (leucine, isoleucine, and valine) in LE adipose tissue versus the non‐LE arm (Figure 4A). To compare the reporter metabolite predictions with the actual metabolite content of the tissue, we performed metabolomics from the frozen adipose tissue samples (Figure 4B). Overall, we did not observe major differences in the concentrations of the analyzed metabolites between the non‐LE and the LE arms. The only significant difference was in valine concentration, which was also predicted to be reduced by the reporter metabolite analyses. We then returned to the RNA sequencing data and checked the expression levels of branched‐chain amino acid transporters. Out of the three transporters *SLC6A15*, *SLC6A14*, and *SLC6A19* that are important for valine metabolism, we observed that *SLC6A15* was one of the most downregulated genes in our RNAseq dataset. These results were further validated by qPCR using the subcutaneous adipose tissue samples from breast cancer surgery‐related LE patients (Figure 1D).

### 3.5 Lipidomics results

**FIGURE 5:**
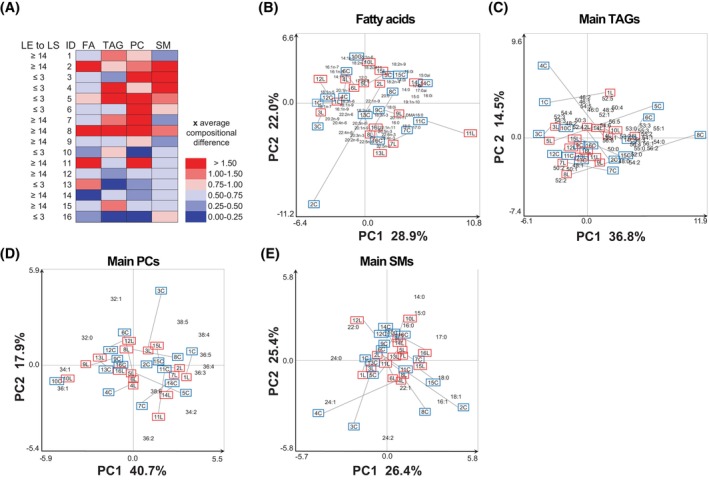
Fatty acid and lipid species profiles of lymphedema arm compared to control (non‐LE) arm. (A) Duration of lymphedema and compositional difference of the two arms of each individual (numbered 1–16) in the PCA scores using fatty acid (FA), triacylglycerol (TAG), phosphatidylcholine (PC) or sphingomyelin (SM) profile data (mol%) as loadings. The degree of the difference was normalized to the average compositional difference of the two arms in the 16 individuals and illustrated in heatmap format (similar composition = 0, average difference = 1, and the largest compositional differences between the two arms got values >1.5). The map was derived from PCA analysis of the (B) FA, (C) main TAG species, (D) main PC species, and (E) main SM species. LE to LS = years from the start of lymphedema to liposuction; ID = ID‐numbers of the individuals in the PCA plots B–E. Variable abbreviation: [total number of acyl chain carbons]:[total number of double bonds]. In addition, for FA, the double bond positions were defined by the n‐x system, and iso‐ and anteiso branches were marked by i and ai, respectively. Plasmalogen‐derived alkenyl chain derivatized to dimethyl acetal was marked with DMA. On the PCA biplots, the samples were marked with the ID and letter C = control (non‐LE) arm (inside blue frame) or L = LE arm (inside red frame).

To analyze the effect of LE on adipose tissue lipid species composition, we next performed untargeted lipidomics on the LE cohort with mass spectrometry and FA profiling by gas chromatography. We were able to detect the main species of TAG, PC, and SM, and the FA profile of total lipids (Figure 5). The main finding was that the lipid species and FA profiles showed large variation between different patients but not consistently between LE versus non‐LE arms, suggesting that the other factors (such as diet, lifestyle, etc.) have a bigger role than the disease in modulating the lipid and FA composition of the adipose tissue (Figure 5A–E). When a relatively large compositional difference between the two arms was found, the patient had either short‐ or long‐term LE (Figure 5A). In several of these individuals, a large difference between the arms in the composition of storage lipid (TAG) was accompanied by a large difference also in membrane lipids (PC and SM). Since the individual FA or lipid species responsible for the compositional difference varied between the patients (the LE samples had shifted from their non‐LE pairs to different directions on the PCA plots), the potential metabolic aberrations behind were individual.

## 4 DISCUSSION

The multi‐omics approach used in this study provides a detailed analysis of the molecular phenotype of LE‐induced adipose tissue. The functional genomics analyses demonstrated that inflammatory and angiogenesis pathways are induced in the edematous adipose tissue compared to the non‐LE arm, whereas pathways related to epidermal differentiation, cell–cell junctions, and water homeostasis were repressed. Interestingly, the gene expression changes in LE‐induced adipose tissue expansion were highly different from the ones found in the subcutaneous adipose tissue in patients with obesity. However, the metabolomes and lipidomes did not differ much between the adipose tissues of LE and the non‐LE arm, but instead, the interindividual variation was much larger. This indicates that the subcutaneous adipose tissue composition is largely determined by other factors (e.g., diet) than LE or its duration.

The whole‐genome transcriptomic analysis revealed that the expression of more than 1400 genes underwent changes in the adipose tissue between the affected and non‐affected arm. This shows that the LE‐induced adipose tissue significantly differs from the same patient's non‐LE adipose tissue. Moreover, we found certain differences between the pathways regulated in short versus long‐term LE as we found a considerable number of genes unique for each group. We also observed that the affected pathways were somewhat different between the short‐ and long‐term LE patients, indicating that the edema phenotype (angiogenesis pathways upregulated while water homeostasis pathway downregulated) is induced early during the disease development and extracellular matrix remodeling is more dominant in the later stages. The downregulation of several keratins in the LE arm might come from the closeness of the skin in the non‐LE arm with a lower amount of adipose tissue.

In both LE and obesity, there is a marked expansion of adipose tissue; however, the nature of this growth proved to be highly different. This means that the LE‐related adipose tissue is unique, both when compared to healthy adipose tissue and to expanded adipose tissue in obesity; it should therefore be treated differently. In clinics, obesity is a risk factor for developing lymphedema after the surgery, which is caused by the excessive amount of adipose tissue that further impairs lymph transport in the affected arm.^50^, ^51^ We have also found that adipocytes in lymphedematous arms and legs are larger than in the nonaffected extremity.^43^ This is in line with the finding that over time larger adipocytes are less insulin sensitive, which we find in the transcriptomics data. It should be noted that some differences in LE‐ and obesity‐induced adipose tissue growth might come from the different adipose tissue depots studied (abdominal vs. arm subcutaneous adipose tissue). However, it has been shown that there are only minor differences in the protein expression between abdominal and femoral subcutaneous adipose tissues.^52^

The finding that the metabolomic and lipidomic profiles of the adipose tissue in the lymphedema arm did not differ much from those of the healthy arm was unexpected. Our findings highlight that systemic effects, such as lifestyle, on adipose tissue lipidomic and metabolomic composition are greater than the disease effect, although the transcriptomic landscape was markedly affected by LE. Since the lipid turnover in adipose tissue is on average only 1–4 years,^53^ the pathways of lipid metabolism, adipocyte differentiation, and insulin responses, that were downregulated in the arms after having LE for ≥14 years, had enough time to modify the adipose tissue FA and lipid compositions. Nevertheless, no consistent metabolome or lipidome differences associated with the disease were found between the LE arms and their pairs (except for valine, discussed later). This suggests that the influence of local differences in the metabolic pathways is masked by a similar supply from the systemic pool via circulation.

In most of the studied patients, the lipidome variation within the patients (between diseased and healthy arms) was smaller than the variation between the patients. In addition, the qualitative differences between the two arms were variable in different patients. The only metabolite that was found to be significantly changed was valine, a branched‐chain amino acid. Also, other branched‐chain amino acids leucine and isoleucine were predicted to be downregulated based on the reporter metabolite analysis, but these amino acids were not detected by the metabolomics platform used in our study. Interestingly, one of the transporters for valine, *SLC6A15* was significantly repressed at the mRNA level, indicating that branched‐chain amino acid metabolism seems to be changed in the LE adipose tissue. These amino acids have been related to obesity‐induced type 2 diabetes,^54^ pointing to a novel direction for further studies in LE and related adipose tissue growth. Interestingly, our finding in LE goes in the other direction, as increased levels of branched‐chain amino acids are found in obesity.

It is likely that the differences we found in the transcriptomics results are caused mainly by other cells than adipocytes. For example, blood and lymphatic endothelial cells, macrophages, and fibroblasts have a large impact on adipose tissue function and phenotype. An injurious immune response after the surgery and radiation, combined with insufficient reparative immune response, has been proposed to be a major driver for lymphatic vascular dysfunction leading to adipose deposition and fibrosis.^55^ Mechanistic insights have been obtained from the modified mouse tail lymphedema model, where inflammation was shown to precede adipogenesis.^56^ Furthermore, leukotriene B4 antagonism by ketoprofen has been shown to effectively ameliorate experimental lymphedema in mice.^57^

A recent study suggested that adipose tissue in LE patients has increased basal lipolysis and cytokine production compared to adipose tissue from healthy control subjects.^58^ A major strength of the present study is the comparison of LE adipose tissue to healthy adipose tissue of the same patient. We further identified patients with short‐ and long‐term disease, allowing us to compare the adipose tissue responses in earlier versus prolonged phases of the disease. We also had the opportunity to compare the findings to a large dataset of female subjects with and without obesity. As a limitation, we were able to run the omics studies in a limited number of patients. For validation of the possibility of using, for example, *CETP*, which was the most upregulated gene, as a biomarker for LE, it would have been useful to have serum samples from these patients. Thus, further studies are needed to address if CETP protein could serve as a serum marker for those breast cancer‐related surgery patients, who are likely to develop LE and related adipose tissue accumulation.

This study provides the first comprehensive map of transcriptional changes that change during LE development over time. An increased understanding of the molecular characteristics of LE‐induced adipose tissue expansion may provide new avenues for the development of novel therapies for LE patients.

## AUTHOR CONTRIBUTIONS

S.K., K.A., and R. Kivelä conceptualized the study. S.K. and R. Kivelä performed investigation and wrote the original draft of the manuscript. H.B. provided the patient samples. S.K., S.L., C.Z., R. Käkelä, A.M., and R. Kivelä contributed to data curation and data analysis. S.K., S.L., C.Z., R. Käkelä, and R. Kivelä contributed to methodology and data visualization. S.L., C.Z., R. Käkelä, A.M., H.B., and K.A. reviewed and edited the manuscript. M.R.T., H.B., and K.A. provided resources. K.A. provided supervision and acquired funding. All authors read and approved the final manuscript.

## DISCLOSURES

The authors declare that they have no conflicts of interest.

## Supporting information


Table S1.

Table S2.

Table S3.

## Data Availability

Data are available upon reasonable request. The differential gene expression comparisons of LE versus non‐LE arms in short‐ and long‐term LE can be found in Table S2. A list of genes that are uniquely regulated in LE or obesity as well as commonly regulated genes in both conditions can be found in Table S3.
