# Supplementary material for: Multi‐omics characterization of lymphedema‐induced adipose tissue resulting from breast cancer‐related surgery
Source: FASEB J. 2024 Oct 12;38(20):e70097. doi: 10.1096/fj.202400498RR (PMC11580717; doi:10.1096/fj.202400498RR)
Supplement: Supplementary file 1 — Table S1. [file FSB2-38-e70097-s002.docx]

**SUPPLEMENTAL INFORMATION**

**Multi-omics characterization of lymphedema-induced adipose tissue resulting from breast cancer-related surgery**

Sinem Karaman, Satu Lehti, Cheng Zhang, Marja-Riitta Taskinen, Reijo Käkelä, Adil Mardinoglu, Håkan Brorson, Kari Alitalo, Riikka Kivelä

**SUPPLEMENTAL TABLE**

**Table S1.** Primer sequences used for quantitative real-time PCR.

| **Gene** | **Sequence (3ʹ-5ʹ)** | **Reference** |
| --- | --- | --- |
| *36B4* | Forward: CAGATTGGCTACCCAACTGTT | PrimerBank ID:  49087144c3 |
|  | Reverse: GGAAGGTGTAATCCGTCTCCAC |  |
| *ABCG1* | Forward: ATTCAGGGACCTTTCCTATTCGG | PrimerBank ID: 46592955c1 |
|  | Reverse: CTCACCACTATTGAACTTCCCG |  |
| *CETP* | Forward: GGCCAAGTCAAGTATGGGTTG | PrimerBank ID:  169636438c1 |
|  | Reverse: ACAGACACGTTCTGAATGGAGA |  |
| *DCD* | Forward: GAAGACCCAGGGTTAGCCAGA | PrimerBank ID:  91208431c1 |
|  | Reverse: GCTCCTTTACCCACGCTTTCT |  |
| *SLC6A15* | Forward: AACTGGGCGGGATTGGATTTG | Self-designed using NCBI Primer Blast, detects variants 1,2,3, and X1 |
|  | Reverse: GGTGGCAGAACTTTGTTCACATT |  |
| *VCAM* | Forward: GGGAAGATGGTCGTGATCCTT | PrimerBank ID:  315434270c1 |
|  | Reverse: TCTGGGGTGGTCTCGATTTTA |  |
| *VEGFC* | Forward: GAGGAGCAGTTACGGTCTGTG | PrimerBank ID:  19924300c1 |
|  | Reverse: TCCTTTCCTTAGCTGACACTTGT |  |
